# Crawling Exercises for Scoliosis

**Published:** 1920-08-21

**Authors:** 


### CRAWLING EXERCISES FOR SCOLIOSIS.

Mr. E. B. Clayton, M.B., B.C., Director of the School of Massage, S.R.E., and Medical Electricity, King's College Hospital, gave an interesting lecture on this subject to the recent Conference of the Incorporated Society of Trained Masseuses on June 26.

The special crawling exercises, which were excellently performed by the patients under treatment at the hospital, were worked out by Dr. Clayton, acting upon the method of Dr. Klapp, Berlin. Dr. Klapp had based his crawling exercises on observations made by him on animals. The object of the exercises, Dr. Clayton explained, was to improve the mobility of the spine and also strengthen the back, shoulder, and hip muscles. In addition to these crawling exercises it was necessary to give educational exercises with a view of maintaining a good posture, and in some cases Swedish Remedial Exercises were given in place of other mobility exercises.

The advantages claimed for crawling exercises were, mainly:

First, the child did not tire so readily as in the upright position. Secondly, the horizontal position allowed more mobility of the thorax and spine, and gave more room for the thoracic organs. Further, it tended to make a flat chest deepen. Thirdly, in the horizontal position postural curves disappeared. With partially fixed curves the back was longer than in the vertical position. Fourthly, the active correction of curves was better than passive, because the muscles were at the same time strengthened. Other advantages were that fewer assistants were required, the child, after being taught, could practise crawling at home three or four times daily; also children liked the crawling exercises.

### Principles of Crawling Exercises.

In speaking of the principles of these exercises, Dr. Clayton referred to a horse taking a long stride forward with the hind leg; it would be noticed, he said, that in so doing the spine would curve with the convexity to the opposite side. Similarly a curve would appear in the dorsal region when one of the front legs was moved forward (convexity to the same side). This curve was increased if the head was turned away from the forward leg. Two curves were used in the performance of the crawling exercises, the C curve and the S curve. The first-named curve was caused in a child's back by the child kneeling with one knee and hand together, and the other leg and arm stretched. By turning the head away from the stretched arm the curve was increased in the dorsal region. S curves were obtained by extending the opposite arm and leg.

### Points of Special Crawls.

The Kyphosis Crawl.—The patient kneels with thighs vertical or sloping forward from knees to hip, and chest on the ground, head extended, and arms stretched above the head. One leg is then raised, with knee straight and brought down in front of the other leg, which in turn is raised also. The arms slide forward along the ground as the patient progresses. The chest must be kept low all the time.

The Kypho-Lordosis Crawl.—The patient commences with the kyphosis crawl, but, instead of raising a leg, draws the arms backwards and progresses by leaping forward between the hands.

High Crawl for Mobility or C Curve.—The knee should be advanced on the convex side, and the hand of the same side placed to outer side of ankle. The leg on the concave side should be extended and the arm of the same side swung over the head, the body then raised as high as possible at the hips. When in position, the trunk, in sagittal plane, is turned so that the back faces one way and abdomen the opposite.

